# The critical pharmaceutical situation in Sudan 2023: A humanitarian catastrophe of civil war

**DOI:** 10.1186/s12939-024-02103-9

**Published:** 2024-03-13

**Authors:** Lina Hemmeda, Angad Tiwari, Barakat Olajumoke Kolawole, Fathima Shehnaz Ayoobkhan, Kainat Fatima, Moshi Moshi Shabani, Mrinmoy Kundu, NagaSpurthy Reddy Anugu, Riya Mary Richard, Danya Ibrahim, Khabab Abbasher Hussien Mohamed Ahmed

**Affiliations:** 1 Faculty of Medicine, University of Khartoum, Khartoum, Sudan; 2 Maharani Laxmi Bai Medical College, Jhansi, India; 3 Department of Community Medicine, Ahmadu Bello University, Zaria, Nigeria; 4 Georgia Cancer Center, Augusta, GA United States; 5 Bahauddin Zakariya University, Multan, Pakistan; 6 National Institute for Medical Research, Mbeya, Tanzania; 7 Institute of Medical Sciences and SUM Hospital, Bhubaneswar, India; 8 MediCiti institute of Medical Sciences, Hyderabad, India; 9 Ivane Javakhishvili Tbilisi State University, Tbilisi, Georgia

## Abstract

The situation in Sudan have deteriorated since the ongoing war outbreak in April 2023. This article sheds light on the pharmacological status in Sudan in terms of shortage of supply, rising demands, and regulatory issues. The ongoing civil war has acutely impacted the dilapidated pharmaceutical status of Sudan, patients have suffered from the paucity of medical services forcing an out-of-control rise in underreported morbidity and mortality. To mitigate this uprising issue, an increase in stakeholder communication is crucial to deal with this national threat and establish a system for reporting the shortage.

## Introduction

Sudan is a low-income country in northeastern Africa [1], that has been influenced by a variety of internal and external factors, such as political and tribal conflicts, economic sanctions, and a lack of economic diversification. There are prospects for economic growth and development owing to the country’s abundant natural resources, which also include agricultural land, minerals, and oil [2]. Sudan’s economy is currently in a critical situation as a result of a number of economic and political issues, which were worsened by the COVID-19 pandemic, and caused a further drop in economic prosperity and a rise in inflation. Sudan’s heavy national debt is one of the main issues that is expected to rise between 2023 and 2028 by 254.7 billion U.S. dollars (+ 266.31%) reaching 350.31 billion U.S. dollars in 2028 [3]. As a consequence, the nation has had trouble obtaining foreign financing, which made economic investment scarce [4]. The pharmaceutical market is consequently affected by this economic crisis.

On the 15th of April 2023, the conflict of the ongoing civil war -between the Sudanese army and Rapid support forces (RSF)- resulted in a stormy acute deterioration in safety, basic needs, and health services for more than three months. Sudan has been witnessing a looming shortage of medicines and medical supplies, which is an impediment to the provision of healthcare services, taking into account that the pharmaceutical crisis has resulted in huge security, political and economic repercussions for the country. This article sheds light on the present pharmaceutical crisis in Sudan, outlines its potentially rooted causes, and analyses the contributing elements to this troubling issue. Also, it includes some recommendations that we believe could serve to ease the situation.

## Pharmaceutical supply chain under the financial strain

Drug supply in Sudan is provided by the public and private sectors. In the public sector, procurement is maintained through the National Medical Supply Fund (NMSF) and the National Medicines and Poisons Board (NMPB), which manages and inspects storage facilities, documents medicinal information, and promotes best practices. The private sector supplies medicines through local manufacturing factories and direct importation from international manufacturers [5, 6]. After the war ignited, the situation broke down; hospitals and renal dialysis centers were shut or destroyed as well as the medications import stopped in the first month due to the airports shut down which affected patients with acute and chronic conditions. The humanitarian aid including medical supplies has helped to some extent, but some of it is not delivered because of the roguery and unsafety [7, 8].

The local medicine production is quite low (5%), the National Medicines and Poisons Board (NMPB) reports the pharmaceutical market (TPM) contribution of the public and private sectors at 24%, and 76% respectively, which is estimated with total of 650 million USD [5]. Due to the difficulty in obtaining raw materials, a significant number of local pharmaceutical manufacturers have closed. Additionally, in half of their manufacturing capacity, 19 out of the 27 pharmaceutical manufacturers in Sudan are reported working, which covers 45% of the nation’s consumption [9].

Almost 45% of the Sudanese population is below the poverty line, and around three-quarters of the population pays out of pocket for their health care [10], which reveals the poor funding for health services [11]. Patient out-of-pocket expenses limit access to chronic disease medications because of the financial burden. Moreover, professional Pharmacists Association of Sudan believes certain drugs cost more than the minimum wage; Sudan minimum wage in April 2020 − 425 SDG- is lowest than the cost required for diabetic medicine [10], and the price of generic drugs is three times the international reference price, and antibiotics like Ceftriaxone injection costs approximately 14 days’ wage whereas Diclofenac costs around 5 days’ wage [12]. Overall, antibiotics, pediatric, and diabetic prescription costs are all unaffordable [10]. The aforementioned facts highlight that medicine’s accessibility is governed by affordability, in most developing countries like Sudan.

The average stock-out duration in Sudan is 18 days, with Khartoum having the longest (59 days) [9]. However, during covid-19, the situation was further complicated by the high demand for antibiotics, antimalarial, and vitamin supplements, which are supplied mostly by the private sector at high unaffordable costs to families that are below the poverty line [13]. Additionally, there has been a shortage of penicillin, antivirals, and antifungals for more than 40 days, which adversely affects patient outcomes [14].

## Causes of the shortage in Sudan before the civil war 2023

One of the most important issues facing low- and middle-income countries (LMICs) is the inadequate availability of medications. More than two billion people are reportedly without access to medications. Medicine access is a fundamental human right. Human rights are infringed when people are denied access to medications for their ailments, especially necessary medications. In order to guarantee that necessary medications are accessible, the World Health Organization (WHO) has recommended the creation and implementation of a national pharmaceutical policy. For pharmaceutical suppliers in the public and private sectors, this policy is “a commitment to a goal and a guide of action”. By addressing every facet of the pharmaceutical industry, its goal is to guarantee the public’s appropriate usage of important, high-quality medicines and their accessibility [36, 37].

In Sudan drug shortages have become a growing problem limiting patients’ access to essential drugs and the provision of healthcare services.

These shortages have many different root causes, which can be divided into three primary groups: supply issues, demand concerns, and regulatory issues. According to a study conducted in Sudan prior to the war, patients at public health facilities had limited access to necessary medications because of high costs and pervasive poverty. The study also evaluated patients’ perceptions of the acceptability and accommodation of the public facility. Certain medicine categories—including antimalarials—have reasonable drug prices, but there is still room for improvement in the affordability of numerous other drug categories, like analgesics and antibiotics, whose prices are higher than the global average. PHC’s capacity does not seem to be meeting the needs of the population overall [35]. But the conditions seem to become far worse after the war.

### Supply issues

In low-income countries like Sudan, supply chain disruptions are frequent causes of medicine shortages [15]. The shortage of raw ingredients, production difficulties, the lack of investment in the pharmaceutical sector, and inadequate infrastructure are frequently the result of breakdowns in the drug supply chain. Also, the gap to the private sector has been widened by a shortage of skilled labor, non-generation, and the low number of jobs in hotspots of the public sector’s medicine chain and points of service i.e., only 26% of pharmacists work in the public sector [15]. Sudan imports about 40% of its medicines. Various challenges the country has faced over the last decade in importing medicines involve problems with trade sanctions, inappropriate pricing policy, poor manufacturing, corruption, and bad governance [5, 16, 17]. Furthermore, these factors have been related to the decline in the local currency relative to the United States Dollar (USD) in Sudan.

### Demand issues

Up until the middle of the 1980s, medications were sold to the general population at subsidized prices. After 1992, the new policy has led to the privatization of drugs [18], the high cost of medicines and lack of health insurance coverage makes it difficult for many Sudanese people to afford the drugs they need till this moment. Medications demand has been maximized due to the rapidly growing population, the rising burden of chronic diseases, the government’s limited resources, and poor funding for the healthcare sector. Other causes include the irrational use of medicines, patients’ low level of education, and failure to follow prescription instructions resulting in wasted medication, subpar results, and increased demand [19]. In addition to the unethical drug advertisement by pharmaceutical companies and prescribing practices [20].

### Regulatory issues

The lack of an effective regulatory framework for the pharmaceutical industry can lead to the prevalence of substandard, counterfeit, harmful, and ineffective medicines on the national markets and international commerce. The Sudanese government has designed several strategies to safeguard the public against low-quality medicines, it is expected that the pharmaceutical departments will be supplied with materials and qualified personnel to effectively perform their duties. For instance, whenever the pharmacy is open for business, a superintending pharmacist is permanently registered with the Sudan Medical Council and licensed to supervise and eliminate counterfeit medications in pharmacies [21]. It is challenging for the government to respond to market changes in the absence of a trustworthy system for monitoring drug quality, pricing, and availability. According to a recent unpublished post-marketing surveillance, 16% of private company (registered product) samples and 35% of Central Medical Supplies Public Organization (CMSPO) samples acquired from various pharmacy shops failed to pass the quality test [22].

In addition to the insufficiency of local drug plants [23], In 2016 the Sudanese government approved new policies, which resulted in a significant rise in the price of drugs or [23] by preventing Sudanese drug manufacturing companies from buying their raw materials i.e., they had to buy from third parties [24]. By the end of the following year, local drug companies stopped selling their drugs due to massive fluctuations in the rate of dollar exchange price. The Lack of price control is another major challenge that affects the distribution of medicines in Sudan. The NMPB was not able to control the prices of imported medicines and 23% of selected imported medicines approved by the agency were ten times more expensive than the internationally recognized reference price [6, 10, 25]. The labor strikes in local drug manufacturing companies further highlight the tension in trying to regulate medicine prices in a fair and effective manner. The government’s adaptation of a policy of price liberalization in 2018 has had unintended consequences [18].

## The exacerbation during COVID-19

Sudan as a country has been crippled by the drug shortage which was further exposed during the COVID-19 period.

Since decluttering the genetic sequence of covid-19 to make its antidote, the world has gone through huge suffering. Apart from a dramatic loss of human life worldwide, the global pandemic has been an unprecedented challenge to public health and Sudan has been no exception. The shortage of drugs, medicine, and medical supplies has been compounded by the rise in COVID-19 cases [26]. To combat this a nationwide lockdown was implemented, similar to measures taken globally. Unfortunately, the lockdowns had a negative impact on the availability of pharmaceutical services, especially for patients in rural areas distant from the capital city of Khartoum [26]. Sudan is facing numerous challenges as it moves ahead during this period of transition. The government’s response to the COVID-19 pandemic has been hampered by the country’s deteriorating economic condition followed by a significant loss of human lives. It is imperative that immediate steps are taken to ensure proper access to pharmaceutical services and establish a reliable and efficient supply chain for medicine in Sudan.

### The deteriorating pharmaceutical situation during the war

The ongoing conflict in Sudan and looming danger have exacerbated the Supply shortage; through the deflection of supply transportation, difficulty in reaching healthcare services, and priority given to the military, which makes it challenging to provide medical services including medications for patients with acute and chronic conditions [27]. Healthcare institutions have suffered significantly, for instance, in the Yemeni conflict [38]. There was destruction of equipment and medication. There have been reports of sharp price increases and shortages of medications [39]. Millions of Yemeni citizens had a serious issue with access to medications. There were high rates of death and morbidity because people could not afford basic medical care, medications for chronic conditions like diabetes, hypertension, and cardiovascular disease, or supplies for diseases that frequently broke out, such cholera and dengue fever [40].

The health crisis scope in Sudan incorporates the rising number of acute cases (4647 injured civilians) as well as patients with chronic diseases such as hypertension, diabetes, and renal failure patients. More frustration has been generated with 66% of Khartoum hospitals being out of service [28], the electrical supply cut has damaged insulin injections and vaccines, and the medication import has stopped. This acute despair to obtain treatment for patients of all ages has affected females in different ways; in reaching obstetric care as well as the inaccessible birth and preventative medications for pregnancy and sexually transmitted infections (STDs) due to the increasing number of rape cases [29, 30]. In addition, the polluted drinking water has aggravated the situation -the rising need for medication due to the various morbidities- and made the pharmaceutical situation collapsing harder with the urgent need for interventions [31]. Looking into the bright side, this disastrous situation unraveled the cooperation of Sudanese civilians, as a group of pharmacists created a virtual group on various social media platforms. The group was named “Wafra”, an Arabic word that means “abundance”, pharmacists from around the country gathered there to announce the newly arrived drugs and notify citizens with the pharmacies where they would be sold. As well, still deteriorating but international aid has helped to mitigate the brunt of the damage temporarily [28, 30, 32].

## Conclusion

The availability and affordability of essential medicines remain top priorities during this devastating civil war. To address drug shortages, evidence from all countries, particularly Sudan, is required for comparison and development of global mitigation strategies.

## Recommendations

International regulatory authorities must collaborate to create a global mitigation plan with a consistent definition and strategy involving low and middle-income countries. Furthermore, steps should be taken at the national level to develop a proactive system for advance notification, reporting, and tracking of drug shortage information. Effective policies should be put in place to: build a strong supply chain, encourage manufacturers to use valued quality systems and manufacture medicines that are at risk of shortage. Training health professionals to educate the public will minimize health problems burden. It is critical to involve all stakeholders at the national and international levels in order to deal with this global threat at all economic levels. Medication subsidies should be considered by stakeholders as a potential strategy that will significantly reduce patients’ drop to out-of-network services and its subsequent health consequences in Sudan.

To deal with drug shortages, most high-income and middle-income countries have proposed various strategies. International and national organizations, such as the World Health Organization (WHO), and the International Pharmaceutical Federation (FIP), are heavily involved in taking initiatives, providing information, and establishing guidelines to alleviate the drug shortage situation.

However, because the problem is still present and has been ignored in the majority of low and middle-income countries, there is a need for ever-growing, universal, and updated versions of the strategies to bring the issue to a halt globally.

Sudan is under multiple crises; the ongoing civil war struck Sudan into a deeper calamity in many ways. The scarcity of medicines, particularly lifesaving drugs, poses a serious public health threat. Urgent efforts should be made to ensure immediate access to pharmaceutical services and a sustainable steady supply of medicines as well. Establishing humanitarian pharmaceutical supply channels to avoid the consequences of an impending crisis as well as authorizing effective policies on drug importation, production, pricing, and distribution. Long-term solutions and efficient pharmaceutical policies should be implemented as part of approaches to strengthen health systems, with medicines and health technologies serving as one of their building blocks. Some major points to be considered are:

- Managing the Current Drug Shortage.

1. Restrictions on current stock usage.
2. The use of products with minor flaws.
3. Extension of the expiry date.
4. Redistribution of available inventory.
5. Construction of the medical expert platform.
6. Wastage administration.

- Operational Enhancement:

1. Increase stakeholder communication.
2. A system for reporting and tracking drug shortages.
3. An increase in generics manufacturers.
4. Improved quality control system.
5. Contacting a wider range of suppliers.

These suggestions and remedies are comparable to those that were previously discussed and demonstrated to be effective in Syria, where researchers discovered that four complementary international legal frameworks could be mutually reinforced to enhance (but not always remedy) the country’s access to medical care during a war. investigates the frameworks of international criminal law, international drug control law, international humanitarian law, and international human rights law. Each of these legal frameworks has a specific area of emphasis [33]. Similar practices also demonstrated success in a Yemeni study, which recommended the application of four key protocols [34].

## Data Availability

All data relevant to the study are included in the article.

## Declarations

### Patient and public involvement

Patients and/or the public were not involved in the design, conduct, reporting, or dissemination plans of this research.

## References

[1] New World Bank country classifications by income level. 2022–2023. https://blogs.worldbank.org/opendata/new-world-bank-country-classifications-income-level-2022-2023. Accessed 18 July 2023.

[2] What Are The Major Natural Resources Of Sudan? 2021. https://icetonline.com/what-are-the-major-natural-resources-of-sudan/. Accessed 18 July 2023.

[3] Sudan - national debt. 2028. Statista. https://www.statista.com/statistics/531953/national-debt-of-sudan/. Accessed 18 July 2023.

[4] Prioritization of Medicine Importation by the Private Sector in Sudan. Evidence from a Data Analysis, 2012–2015 - PubMed. https://pubmed.ncbi.nlm.nih.gov/32653861/. Accessed 18 July 2023. 10.1016/j.vhri.2019.11.007 32653861

[5] Rebuilding Sudan’s. health system: opportunities and challenges - PubMed. https://pubmed.ncbi.nlm.nih.gov/31954447/. Accessed 18 July 2023.

[6] Velpandian V, Elangovan S, Agnes L, Musthafa M. Clinical Evaluation of Justicia tranquebariensis L. In the Management of Bronchial Asthma. American Journal of Phytomedicine and Clinical Therapeutics. 2014. https://www.semanticscholar.org/paper/Clinical-Evaluation-of-Justicia-tranquebariensis-L.-Velpandian-Elangovan/e149394c69322f29386685e83659f66aecadde02. Accessed 18 July 2023.

[7] Sudan’s war. exacts deadly toll on dialysis patients, leaves bodies rotting| Reuters. https://www.reuters.com/world/africa/sudans-war-exacts-deadly-toll-dialysis-patients-leaves-bodies-rotting-2023-06-14/.

[8] Sudan: ‘Secure and immediate access’ needed for lifesaving aid, urges Guterres, News. https://news.un.org/en/story/2023/05/1136302.

[9] Pharmacists. ‘Catastrophic’ medicine situation in Sudan - Dabanga Radio TV Online. https://www.dabangasudan.org/en/all-news/article/pharmacists-catastrophic-medicine-situation-in-sudan. Accessed 18 July 2023.

[10] Cheraghali AM, Idries AM (2009). Availability, affordability, and prescribing pattern of medicines in Sudan. Pharm World Sci.

[11] Some Aspects of medicine distribution in Sudan. https://www.heighpubs.org/hps/apps-aid1009.php. Accessed 18 July 2023.

[12] Sefah IA, Ogunleye OO, Essah DO, Opanga SA, Butt N, Wamaitha A (2020). Rapid Assessment of the Potential Paucity and Price Increases for Suggested Medicines and Protection Equipment for COVID-19 across developing Countries with a Particular Focus on Africa and the implications. Front Pharmacol.

[13] Ali G, Abobaker Y, Abdelrahman M, Alfadl A. *20.12.2021 measures set by NMRA submitted to UU winter meeting 13–14 Jan 2022*. 2022.

[14] Healthcare Utilization with Drug Acquisition and Expenses at the National Health Insurance Fund. in Sudan - PubMed. https://pubmed.ncbi.nlm.nih.gov/35455808/. Accessed 18 July 2023. 10.3390/healthcare10040630 PMC9024857 35455808

[15] Availability and Use of Therapeutic Interchange Policies in Managing Antimicrobial Shortages among South African Public Sector Hospitals.; Findings and Implications - PubMed. https://pubmed.ncbi.nlm.nih.gov/31861923/. Accessed 18 July 2023. 10.3390/antibiotics9010004 PMC7168338 31861923

[16] Pharmaceuticals in frontier markets.: Part 3 - Sudan. https://www.diligenciagroup.com/blogs/pharmaceuticals-in-frontier-markets-part-3-sudan. Accessed 18 July 2023.

[17] Drugs WAP. on E. Comparative analysis of national drug policies in 12 countries: second workshop, Geneva, 10–13 June 1996. 1997. https://apps.who.int/iris/handle/10665/63513. Accessed 18 July 2023.

[18] Drug. shortage crisis in Sudan in times of COVID-19 - PubMed. https://pubmed.ncbi.nlm.nih.gov/36101692/. Accessed 18 July 2023.

[19] Shukar S, Zahoor F, Hayat K, Saeed A, Gillani AH, Omer S (2021). Drug shortage: causes, impact, and mitigation strategies. Front Pharmacol.

[20] Mandour ME. 5.5 Country case study: Sudan.

[21] Walker J, Chaar BB, Vera N, Pillai AS, Lim JS, Bero L (2017). Medicine shortages in Fiji: a qualitative exploration of stakeholders’ views. PLoS ONE.

[22] Malik M, Hassali MAA, Shafie AA, Hussain A (2013). Why hospital pharmacists have failed to manage antimalarial drugs stock-outs in Pakistan? A qualitative insight. Malar Res Treat.

[23] Sudan Suffers From Medical Supplies Crisis. https://english.aawsat.com/home/article/2198971/sudan-suffers-medical-supplies-crisis. Accessed 18 July 2023.

[24] Khder SI, Alwakeel A, SaifAldawla A, Ali AA, Kadoma M, Hassan N (2020). Measuring availability and prices of locally Produced and Imported Medicines in Sudan. JMID.

[25] Dabanga. Medics raise alarm on medicine shortages in Sudan. Dabanga Radio TV Online. 2021. https://www.dabangasudan.org/en/all-news/article/sudanese-doctors-raise-alarm-on-medicine-shortages-in-the-country. Accessed 18 July 2023.

[26] Sudan warns of looming food and medicine crisis| The East African. https://www.theeastafrican.co.ke/tea/rest-of-africa/sudan-warns-of-looming-medicine-crisis-3573118?view=htmlamp. Accessed 18 July 2023.

[27] Salih ZM. Doctor who criticised army for diverting aid detained in Sudan. The Guardian. 2023. https://www.theguardian.com/world/2023/may/31/sudan-arrests-doctor-who-criticised-army-for-diverting-aid. Accessed 18 July 2023.

[28] SAPA-USA– Sudanese American Physicians Association. https://sapa-usa.org/. Accessed 18 July 2023.

[29] Strzyżyńska W. Anguish as rape survivors in Sudan unable to access vital medication. The Guardian. 2023. https://www.theguardian.com/global-development/2023/jun/14/anguish-as-survivors-in-sudan-unable-to-access-vital-medication. Accessed 18 July 2023.

[30] Sudan| ReliefWeb. 2023. https://reliefweb.int/country/sdn. Accessed 18 July 2023.

[31] Salih ZM, Michaelson R. Medics in Sudan warn of crisis as health system near collapse. The Guardian. 2023. https://www.theguardian.com/world/2023/may/01/medics-in-sudan-warn-of-crisis-as-health-system-near-collapse. Accessed 18 July 2023.

[32] Donate Now.: Support the Kidney Care Community in Sudan to Overcome Critical Challenges Caused by the Conflict - International Society of Nephrology. https://www.theisn.org/blog/2023/06/06/donate-now-support-the-kidney-care-community-in-sudan-to-overcome-critical-challenges-caused-by-the-conflict/. Accessed 18 July 2023.

[33] Leyh BM, Gispen ME. Access to medicines in times of conflict: Overlapping compliance and accountability frameworks for Syria. PubMed. 2018;20(1):237–50. Available from: https://pubmed.ncbi.nlm.nih.gov/30008566. PMC6039728 30008566

[34] Alshakka M, Ibrahim MIM, Bahattab A, Badulla WFS, Shankar PR. An insight into the pharmaceutical sector in Yemen during conflict: challenges and recommendations. Medicine, Conflict and Survival. 2020;36(3):232–48. 10.1080/13623699.2020.1794287. 10.1080/13623699.2020.1794287 32718201

[35] Hemmeda L, Koko A, Mohamed R, Mohammed YIA, Elabid AOM, Omer AT et al. Accessibility crisis of essential medicines at Sudanese primary healthcare facilities: a cross-sectional drugs’ dispensaries assessment and patients’ perspectives. International Journal for Equity in Health. 2023;22(1). 10.1186/s12939-023-02009-y. 10.1186/s12939-023-02009-y PMC10583350 37848939

[36] WHO. *The World Medicines Situation 2004* World Health Organization (WHO); Geneva, Switzerland: 2004. [(accessed on 18 February 2020)]. Access to Essential Medicines. Available online: http://apps.who.int/medicinedocs/en/d/Js6160e/9.html [Google Scholar].

[37] World Health Organization (2001). How to develop and implement: a National Drug Policy.

[38] Health crisis in Yemen. International Committee of the Red Cross. 2023. Available from: https://www.icrc.org/en/where-we-work/middle-east/yemen/health-crisis-yemen.

[39] Half of all health facilities in war-torn Yemen now closed; medicines urgently needed– UN, UN News. 2017. Available from: https://news.un.org/en/story/2017/03/554212-half-all-health-facilities-war-torn-yemen-now-closed-medicines-urgently-needed.

[40] World Health Organization: WHO. WHO airlifts over 500 tons of essential medicines and medical supplies to Yemen. World Health Organization. 2018; Available from: https://www.who.int/news/item/02-09-2018-who-airlifts-over-500-tons-of-essential-medicines-and-medical-supplies-to-yemen.

